# Normal Pedobarography Values in a Multiracial Community of Malaysia: A Cross-Sectional Study

**DOI:** 10.5704/MOJ.2511.001

**Published:** 2025-11

**Authors:** MN Haidar, A Che-Ahmad, MAS Ayeop, MS Ahmad-Ismani, NAA Nik-Alyani

**Affiliations:** Department of Orthopaedics, Traumatology and Rehabilitation, International Islamic University Malaysia, Kuantan, Malaysia

**Keywords:** foot pressure, plantar pressure, pedobarography, demographic factors, BMI

## Abstract

**Introduction:**

While pedobarography is emerging in Malaysia for foot-related clinical and research use, there are no published normative foot plantar pressure values. This study aimed to identify pressure values and areas of distribution in the normal foot of the multiracial population in Pahang, Malaysia. The differences by ethnic groups, gender, and BMI levels were also investigated.

**Material and Methods:**

In this cross-sectional study, 600 normal feet without pre-existing diabetes mellitus and any lower limb and spine pain were assessed using Emed-q100 pedobarography platform device and EMED/E (Expert) software from January 2018 to December 2018. The data were analysed using SPSS, with descriptive statistics for demographics and foot pressure, Pearson Chi-Square for associations, t-tests for gender differences, and one-way ANOVA for comparisons across ethnicity and BMI groups.

**Results:**

The mean maximum peak pressure (MPP) was measured at 508.98 kPa (SD 164.06) with significant differences by BMI whereby the score was highest in the obese (p<0.001). No significant differences by gender and ethnicity were reported. In terms of peak pressure area (PPA), most (42.2%) were observed in the first metatarsal head and big toe region (1MH&T), followed by second metatarsal head (2MH) (31.5%). For overweight and obese, PPA mostly distributed in 2MH (34.8% and 50%, respectively). Significant differences in PPA were also seen between races. There was no significant difference in the pressure area distribution by gender.

**Conclusion:**

The MPP is affected by BMI while PPA distribution is affected by BMI and ethnicity. These findings can be used as an initial reference for further studies related to orthopaedic clinical applications, particularly involving the Malaysian population with various ethnicities and cultural backgrounds.

## Introduction

Pedobarography is the study of foot plantar pressure, which refers to the force or load exerted by the foot onto the plantar surface of individuals during their regular daily locomotor activities. For decades, its applications have been widely explored in developed countries for various fields and applications, including footwear design, sports performance, biomechanics, and foot-related health management^1-3^.

There are various methods available to measure foot plantar pressure, which may vary in complexity and the level of details provided. Since the first documented static foot-print method in 1925, foot plantar pressure measuring techniques had then evolved to more dynamic technique until the development of plantar pressure measuring devices which can either be the platform or the in-shoe system^4^. At present, with the advancement of technologies, among commonly used methods are pedobarography systems which consist the combination of pressure-sensitive insoles or mats and specialised software for data analysis.

The application of pedobarography is particularly promising for diabetic foot problems, and this holds true particularly for Malaysia whereby the prevalence of diabetes mellitus in this country is relatively high. Numerous studies revealed that plantar foot pressures differ by different demographic and health backgrounds, such as ethnicity^5^, gender and age^2^, diabetic condition^6^, as well as BMI level^7^. Yet, to date, there is no known standard plantar foot pressure values for the multi-ethnicity populations in Malaysia as well as the Southeast Asian region.

Therefore, the absence of local population data, in Malaysia specifically, warrants the need to conduct a case study to provide necessary insights towards developing the standardisation of normal foot plantar pressure and thus beneficial inputs for early diagnosis and intervention for foot-related problems involving the local population. Furthermore, the influence of varying demographic background among the citizens necessitates further investigation. This study was conducted to measure the values and areas of distribution of foot plantar pressure in the normal foot of the Malaysian population who are residing in Pahang, which is a state in the east coast region. The differences in the pressure values and distribution areas by ethnic groups, gender, and BMI levels were also investigated.

## Materials and Methods

In this study, a cross-sectional study design was carried out where the pressure values and areas of distribution of a total of 600 normal feet (300 participants) from the multiracial population in Pahang, Malaysia were examined at a specific time point. The sample size was determined using Cochran’s formula with Finite Population Correction (FPC), considering the adult population size in Pahang. The population is estimated around 1,113,906 with 70% Malay, 15.4% Chinese, and 4.2% Indian^8,9^.

The study participants were recruited by convenience sampling from the orthopaedic clinic of Sultan Ahmad Shah Medical Centre (SASMEC) on a voluntary basis from January 2018 to December 2018. The inclusion criteria for the study participants were local Malaysian citizen, aged 18 years and above, with diverse demographic backgrounds (ethnicity and gender) and currently residing in the Pahang state. On the other hand, people with known history of related health issues which might affect the study outcomes, including diabetes mellitus, spine or lower limb deformity, surgical conditions and other trauma, as well as continuous musculoskeletal pain in the lower limb and trunk areas were excluded from this study. These were taken from clinical history. No investigation was done.

Maximum Peak Pressure (MPP) refers to the highest force exerted on a specific area of the individuals’ foot during their daily activities, such as walking or standing. In this study, MPP was measured to assess the participants’ foot biomechanics and potential pressure-related issues. Peak pressure area (PPA) refers to the specific area of the foot where the highest pressure is concentrated during activities like walking or standing, measured to identify regions that may be prone to stress or injury. This variable was assessed in this study to identify and analyse the regions of the foot most susceptible to high pressure, which could indicate areas at risk for potential stress or injury.

**Fig. 1:**
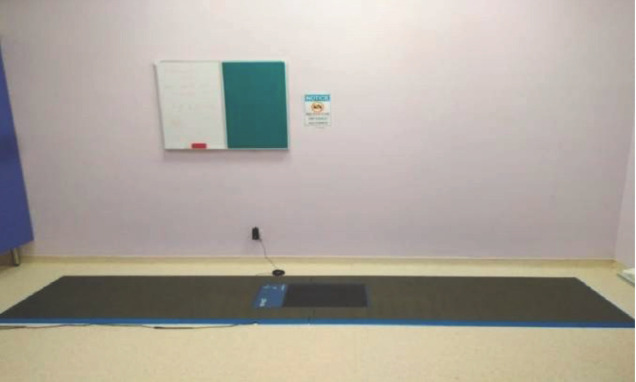
The EMED platform in the pedobarography laboratory.

**Fig. 2:**
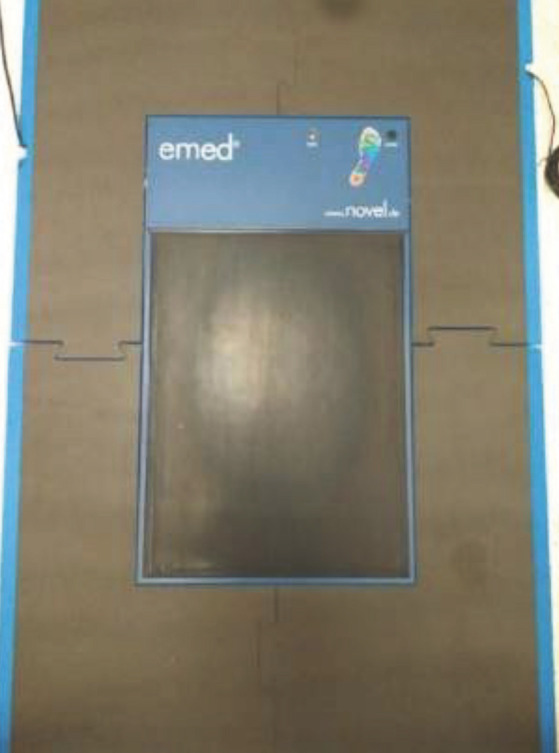
The sensor area located at the middle of the platform.

Foot plantar pressure of the study participants were measured using the EMED-q100 pedobarography platform in combination with the software component, i.e., EMED/E (Expert) software (Fig. 1). In general, this integrated system is utilised to record, process, and evaluate the pressure distribution under the individual’s foot in non-static condition, such as during walking or other weight-bearing activities. The system consists a walking platform with the dimension of 700x403x15.5mm^3^ and a sensor panel with a size of 475x320mm. The sensor area (Fig. 2) was formed by a total of 6080 sensors, with a resolution of 4 sensors/cm^2^ and 100Hz frequency. This specialised system is able to measure plantar pressure within the range of 10-1270 kPa, with a maximum total force of 193,000 N.

Upon obtaining consents from the participants, data were collected at Pedobarography Laboratory in the orthopaedic clinic of Sultan Ahmad Shah Medical Centre (SASMEC). The participants’ weight and height were measured to obtain their BMI level. Prior to the actual data collection, briefings and demonstrations were provided to the study participants pertaining to the correct walking method on the platform. The study participants were provided with few trial sessions to familiarise themselves in walking on the platform.

**Fig. 3:**
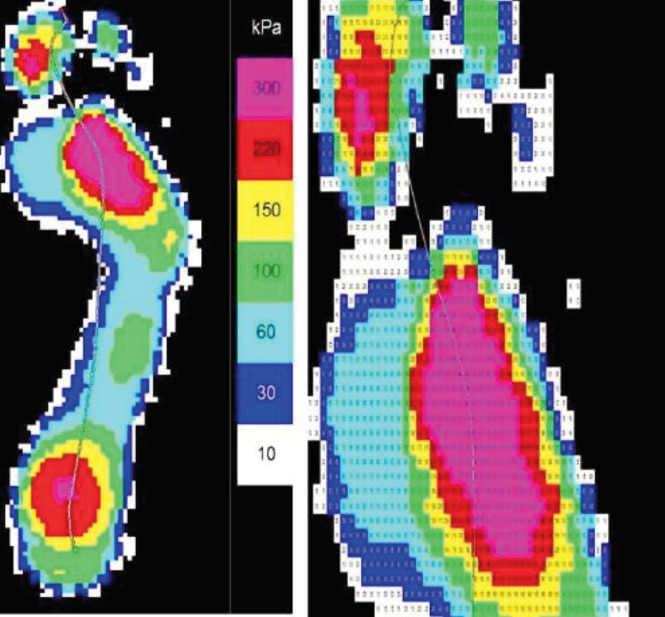
Example measurement results: The highest-pressure value (580kPa) was identified at the pink area at 2MH.

The plantar foot pressure of each study participant was measured using the two-step method from the EMED/E (Expert) system, as outlined by the manufacturer. This was due to the ability of this method to produce results which are equivalent to those of the mid-gait method with five trials. The results obtained through the software indicate the mean peak pressure values and pressure distribution area, as the example shown in Fig. 3.

The primary objective in this study was the measurement of MPP and PPA in the participants’ normal foot conditions. These variables were examined to investigate how foot pressure of the studied samples is distributed across different regions of the foot, and to identify potential areas of high stress that could lead to injury.

In addition to the primary outcome above, the study also examined secondary issues of interest, specifically regarding the effects of demographic factors such as ethnicity, gender, and BMI on the participants’ foot pressure measurements. By comparing these variables across different demographic groups, the study aimed to identify significant variations in foot biomechanics among a multiracial population, such as in studied area in this study.

This study has been approved by the International Islamic University Malaysia Research Ethic Committee (IREC 2018-171). Informed consents were obtained from all participants prior to their inclusion. Data was saved in a password-protected file and can only be accessed by the researchers of this study, protecting the participants’ confidentiality and maintaining data security standard.

The collected data were analysed using IBM SPSS version 24. Descriptive analysis was performed to obtain mean, frequency, and percentage values for demographic data (age, ethnicity, gender, weight, height, BMI, peak pressure scores, and PPA). Pearson Chi-Square tests were conducted to determine significant associations between categorical variables. Independent t-tests compared peak foot pressure by gender, while one-way ANOVA compared ethnicity and BMI levels.

## Results

The participants’ demographic profiles (age, gender, ethnicity, weight, height, BMI group, and mean of plantar foot pressure) are presented in Table I. Majority the study participants were Malay (66%), followed by Chinese and Indian (22.7% and 11.3%, respectively). This represents closely the percentage of ethnicities in Pahang^9,10^. The average age was approximately 33 years old (SD±14.47). Majority of them were female (52.3%). In average, the participants' weight was 65.08kg (±15.46), and their height was 162.96cm (±9.45). In terms of BMI level, 54.7% of the participants had normal BMI. The mean plantar foot pressure value was recorded at 508.98kPa (SD ±164.06). In terms of the distribution of PPA, most (42.2%, n=253) were observed in first metatarsal head and big toe region (1MH&T), followed by second metatarsal head (2MH) (31.5%, n=189). Next, the distribution of PPA were analysed to study the significant differences by several demographic factors. First, the comparison results by gender are presented in Table II. There was no statistically significant difference in the distribution of PPA between male and female participants (p>0.05). Therefore, it can be inferred that gender did not have any statistically significant association with the participants' PPA distribution.

**Table I:** Demographic data (N = 600).

Effect	Frequency (n)	%
**Age (years)**		
Mean (±SD) = 32.85 (±14.47)	n/a	n/a
**Gender**		
Male	286	47.7
Female	314	52.3
**Ethnicity**		
Malay	396	66.0
Chinese	136	22.7
Indian	68	11.3
**Weight**		
Mean (±SD) = 65.08 (±15.46)	n/a	n/a
**Height**		
Mean (±SD) = 162.96 (±9.45)	n/a	n/a
**Body Mass Index (BMI)**		
Underweight	42	7.0
Normal	328	54.7
Overweight	164	27.3
Obese	66	11.0
**Peak Pressure Area (PPA)**		
1MH & 1T	253	42.2
2MH	189	31.5
3MH	90	15.0
4MH & 5MH	41	6.8
2T & 3-5T	2	0.3
HF	25	4.2
**Peak Foot Pressure**		
Mean (±SD) = 508.98 (±164.06)	n/a	n/a

**Table II:** Comparison of PPA by gender (N=600).

				Peak Pressure Area (PPA)							Total	P-value*
		1MH	2MH	3MH	4MH	5MH	1T	2T	3-5T	HF		
Gender	Male	12 (4.2%)	95 (33.2%)	45 (15.7%)	3 (1.0%)	13 (4.5%)	103 (36.0%)	1 (0.3%)	0 (0%)	14 (4.9%)	286	0.617
	Female	10(3.2%)	94 (29.9%)	45 (14.3%)	3 (1.0%)	22 (7.0%)	128 (40.8%)	0 (0%)	1 (0.3%)	11 (3.5%)	314	
Total		22	189	90	6	35	231	1	1	25	600	

Comparison of the PPA by different ethnicity are presented in Table III. The Pearson Chi-Square test revealed a significant association between the areas of distribution and the participants’ ethnicity (p<0.05). The Malay participants recorded the highest percentage at the 1T region (36.1%), followed by 2MH and 3MH (33.6% and 16.2%, respectively). The Chinese participants indicated a similar pattern whereby 1T recorded the highest percentage (38.2%), followed by 2MH and 3MH (33.1% and 8.8% respectively). As for the Indian participants, while the highest percentage was similarly observed at the 1T area (47.1%), this, on the other hand, was followed by 3MH (20.6%) and 2MH (16.2%).

**Table III:** Comparison of PPA by ethnicity (N=600).

				Peak Pressure Area (PPA)							Total	P-value*
		1MH	2MH	3MH	4MH	5MH	1T	2T	3-5T	HF		
Ethnic	Malay	7 (18%)	133 (33.6%)	64 (16.2%)	3 (1.0%)	22 (5.6%)	147 (36.1%)	0 (0%)	0 (0%)	18(4.5%)	396	0.007
	Chinese	11 (8.1%)	45 (33.1%)	12 (8.8%)	1 (0.7%)	9 (6.6%)	52 (38.2%)	1 (0.7%)	1 (0.7%)	4 (2.9%)	136	
	Indian	4 (5.9%)	11 (16.2%)	14 (20.6%)	0 (0%)	4 (5.9%)	32 (47.1%)	0 (0%)	0 (0%)	3 (4.4%)	68	
Total		22	189	90	6	35	231	1	1	25	600	

Next, comparison of the PPA distribution by BMI are presented in Table IV. There was a statistically significant association between both variables (p<0.05). Specifically, those with normal BMI recorded the highest percentage at the 1T region (45.1%), followed by 2MH and 3MH (25.0% and 13.7%, respectively). Meanwhile, those overweight showed the highest occurrence at the 2MH region (34.8%), followed by 1T and 3MH (33.5% and 13.7%, respectively). This was similarly observed for the obese participants who were mostly distributed at the 2MH region (50%), followed by 1T and 3MH (24.2% and 16.7%, respectively).

**Table IV:** Comparison of PPA by BMI (N=600).

					Peak Pressure Area (PPA)						Total	P-value*
		1MH	2MH	3MH	4MH	5MH	1T	2T	3-5T	HF		
BMI	Underweight	0 (0%)	17 (40.5%)	6 (14.3%)	0 (0%)	1 (2.4%)	12 (28.6%)	0 (0%)	0 (0%)	6(14.3%)	42	0.007
	Normal	11 (3.4%)	82 (25.0%)	45 (13.7%)	4(1.2%)	22 (6.7%)	148 (45.1%)	1 (0.3%)	1 (0.3%)	14 (4.3%)	328	
	Overweight	9 (5.5%)	57 (34.8%)	28 (13.7%)	2 (1.2%)	10(6.1%)	55 (33.5%)	0 (0%)	0 (0%)	3 (1.8%)	164	
	Obese	2 (3.0%)	33 (50.0%)	11 (16.7%)	0 (0%)	2 (3.0%)	16(24.2%)	0 (0%)	0 (0%)	2 (3.0%)	66	
Total		22	189	90	6	35	231	1	1	25	600	

Next, independent t-test and one-way ANOVA analyses were run to compare the means of MPP values by different groups of gender, ethnicity, and BMI. The results were summarised in Table V. There were no statistically significant differences in the mean values of peak foot pressure by gender and ethnicity groups (p>0.001).

**Table V:** Comparison of the mean of peak pressure values by gender (N=600).

	Peak foot pressure	Mean	SD	t-stat / *F-stat	p-value*
Gender	Male	506.84	148.71	-0.305	0.760
	Female	510.93	177.11		
Ethnicity	Malay	509.41	167.61	0.186	0.831
	Chinese	503.24	142.32		
	Indian	517.94	184.58		
BMI	Underweight	432.98	147.36	8.428	<0.001
	Normal	492.64	165.89		
	Overweight	542.41	160.64		
	Obese	555.48	145.23		

On the other hand, the mean difference was statistically significant by the BMI factor (F stat = 8.428, p<0.001). In detailed, the obese participants had the highest score compared to the other groups. A further analysis using multiple comparison (Bonferonni) revealed that the peak foot pressure scores of the underweight and those with normal BMI were significantly different from those overweight and obese (p<0.05).

## Discussion

The current study has emphasised key characteristics of plantar pressure distribution among individuals. The mean plantar foot pressure value was recorded at 508.98kPa. In terms of the distribution of PPA, 42.2% were observed in first metatarsal head and big toe region (1MH&T), followed by second metatarsal head (31.5%). The research found no significant variations in mean peak foot pressure by gender or ethnicity; however, there was a significant difference by BMI, with obese people having the greatest mean plantar foot pressure. Ethnicity made a significant effect on plantar pressure area but not the mean peak pressure values. These findings highlight the relevance of ethnicity and BMI in plantar pressure analysis.

Meanwhile, for foot pressure values, the mean peak foot pressure among the participants in this study was 509 kPa (SD 164.06). Mixed findings have been reported in the literature. While our findings were higher than those in some previous studies^2,3,6^, they were lower than those from studies involving healthy participants in other countries^7,10^. Several factors may contribute to these differences, including demographic characteristics such as age, ethnicity, and geographical background, as well as health-related factors. Additionally, differences in plantar pressure measurement devices may have led to variations in reported values^11^.

Regarding peak pressure area (PPA), our study found that plantar foot pressure in normal feet was primarily distributed at the first ray (1MH&T), followed by the second and third metatarsal head areas (2MH and 3MH), which is consistent with previous studies^12,13,14^. For instance, a study involving 353 healthy individuals in Brazil reported that the highest peak plantar pressure occurred in the forefoot region, with mean values of 408.24kPa ± 44.11 for females and 409.03 ± 45.04kPa for males, followed by the hindfoot region^13^. This high forefoot pressure is clinically significant, as it has been identified as a contributing factor to foot pain in various studies^13,15^.

This study observed that BMI significantly influenced plantar foot pressure values and distribution areas. Obese participants exhibited the highest MPP values, and their peak pressure areas were more frequently distributed in the second metatarsal head (2MH) region. These findings align with those of Ahsan *et al*, who reported a correlation between BMI and increased plantar pressure^7^. The role of BMI in altering plantar pressure patterns is further emphasised by Choi *et al* and Skopljak *et al*, who demonstrated the diagnostic value of pedobarography in assessing weight-related biomechanical changes. Choi *et al* highlighted how pedobarography provides objective plantar pressure mapping to aid in diagnosing foot conditions, while Skopljak *et al* reinforced its role in identifying abnormal weight distribution and gait abnormalities^16,17^.

This study observed that BMI significantly influenced plantar foot pressure values and distribution areas. Statistically significant differences in peak foot pressure values were observed across BMI groups, with obese participants recording the highest values. Additionally, a significant association was observed between BMI and PPA, with normal-weight participants exhibiting pressure primarily in the toe (1T) region, while overweight and obese individuals had higher pressure in the 2MH region. The influence of BMI on plantar pressure was similarly observed by Ahsan *et al* who observed the association of higher BMI and body mass on higher plantar pressure and pressure distribution^7^. A similar finding pertaining to the significant differences of the pressure values by higher BMI level was also reported in a previous study^18^.

Additionally, a statistically significant association was found between ethnicity and plantar peak pressure distribution. While all ethnic groups (Malay, Chinese, and Indian) exhibited the highest pressure in the 1T region, differences were observed in other regions of the foot. However, ethnicity did not significantly influence mean plantar pressure values. In contrast, a study comparing Caucasians and Indians reported significant ethnic differences in peak pressure values but not in contact area^19^. Genetic and environmental factors have been suggested to contribute to such differences, as noted by Costello *et al*^20^.

In contrast, this study did not find a significant association between gender and plantar foot pressure values or distribution areas. This finding is consistent with several previous studies, which reported no significant gender-based differences in plantar pressure^13,14^. For instance, Putti *et al* found that while peak pressure values did not differ significantly between males and females, contact area measurements did^14^. This finding reinforces the importance of using norm-referenced plantar pressure measurements in clinical settings^13^.

The findings from this study contribute to the existing literature by establishing normative values and distribution patterns for plantar pressure in a diverse Malaysian population. Clinically, these findings provide a benchmark for assessing and treating foot-related conditions. Additionally, this study adds valuable data to biomechanical research on plantar pressure, particularly regarding the effects of BMI and ethnicity on foot biomechanics.

However, these results should be considered in light of certain limitations. First, there was an imbalance in the racial composition of the study population, with Malay participants far outnumbering those from other ethnic groups. This limits the ability to draw strong conclusions about the effects of ethnicity. Additionally, the study only examined demographic factors such as gender and ethnicity, whereas other important variables such as aging and lifestyle factors (e.g., physical activity levels) were not included. Furthermore, the study relied solely on quantitative data, without qualitative insights into participants' experiences, cultural influences, or lifestyle factors affecting foot biomechanics. The study was only analysing dynamic plantar pressure so it might not be used for static plantar pressure.

For future research, we recommend increasing the representation of each ethnic group to ensure more inclusive and generalisable findings. Additionally, future studies should investigate other demographic and lifestyle factors, such as age and physical activity levels, to provide a more comprehensive understanding of plantar pressures.

## Conclusions

The study determined mean plantar pressure (MPP) values and distribution regions in the normal foot of a multiracial community, in Pahang, Malaysia with an average MPP of 509 kPa (SD 164). BMI and ethnicity have considerable impact on plantar pressure analysis. These findings serve as a reference for normal plantar pressure values in the Malaysian population. Future study should include more study population and more diverse ethnic representation.
